# Circulating lncRNA DANCR as a potential auxillary biomarker for the diagnosis and prognostic prediction of colorectal cancer

**DOI:** 10.1042/BSR20191481

**Published:** 2020-03-27

**Authors:** Xianjuan Shen, Yajing Xue, Hui Cong, Xudong Wang, Zhiwei Fan, Xiaopeng Cui, Shaoqing Ju

**Affiliations:** 1 Laboratory Medicine Center/Research Center of Clinical Medicine, Affiliated Hospital of Nantong University, 20 Xisi Road, Nantong 226001, China; 2 Laboratory Medicine Center, Nantong Tumor Hospital, 48 Qingnian Road, Nantong 226001, China; 3 Medical School of Nantong University, 20 Qixiu Road, Nantong 226001, China; 4 Department of General Surgery, Affiliated Hospital of Nantong University, 20 Xisi Road, Nantong 226001, China

**Keywords:** auxiliary diagnosis, colorectal cancer, DANCR, real-time PCR

## Abstract

Studies have shown that long non-coding RNAs (lncRNAs) play vital roles in the development of cancer, including colorectal cancer (CRC). Our purpose is to validate the diagnostic value of serum differentiation antagonizing non-protein coding RNA (DANCR) in CRC by focusing on its expression and clinical application. lncRNA expression profiles of CRC patients were obtained and analyzed by repurposing the publically available microarray data. Tissue or serum specimens were obtained from 40 patients with primary CRC, 10 patients with recurrent CRC, 40 patients with colorectal polyps, and 40 healthy controls. It was found that DANCR level in the CRC tissue and serum was significantly increased, and serum DANCR expression was decreased in post-operative patients as compared with that in pre-treatment patients and recurrent patients. In addition, serum DANCR expression was significantly correlated with different TNM stages. Correlation analysis of DANCR and other diagnostic indicators showed that the serum DANCR expression level was significantly correlated with CA199 but not with CEA in CRC patients. As for diagnostic efficiency by ROC analysis, the area under the curve (AUC) of serum DANCR was higher than that of CEA and CA199 in CRC group *vs.* colorectal polyp group. Simultaneous detection of DANCR, CEA and CA199 yielded the highest sensitivity and AUC as compared with either of them alone. Taken together, serum DANCR was up-regulated in CRC patients and high expression of DANCR may prove to be a potential biomarker for the diagnosis of CRC.

## Introduction

Colorectal cancer (CRC) is a common human malignancy, ranking as the third leading cause of cancer-related deaths [1]. Despite the major advances in diagnosis and treatment, CRC remains an intractable malignancy with a poor 5-year survival rate partly due to its susceptibility to metastasis, recurrence and drug resistance, as well as the lack of biomarkers for early diagnosis [2]. To develop the novel therapeutic interventions against CRC, it is necessary to find out CRC-associated pathways, underlying action mechanisms and specific diagnostic or therapeutic biomarkers, knowing that the currently available diagnostic methods have more or less defects. For example, colonoscopy is the gold standard for CRC diagnosis but its invasiveness has limited the wider use for large-scale screening. Fecal occult blood testing is not suitable for screening owing to its unsatisfactory accuracy. The existing diagnostic marker CEA also has a limited utility for CRC because of its low sensitivity, especially for early diagnosis [3]. Therefore, an ideal approach for early-stage detection should have high sensitivity and specificity.

Long non-coding RNAs (lncRNAs) are a class of noncoding RNAs length longer than 200 nucleotides, which are defined as evolutionarily conserved non-protein coding transcripts [4]. Dysregulation of several lncRNAs has been reported to be associated with a series of cellular processes that are closely related to cancer progression such as proliferation, apoptosis and migration [5–7]. Ectopic expression of lncRNAs also affects signaling pathways that are associated with CRC etiopathogenesis [8].

To identify differential lncRNA/mRNAs between CRC and normal samples, GSE126092 microarray dataset was downloaded from the Gene Expression Omnibus (GEO) website, showing that lncRNA differentiation antagonizing non-protein coding RNA (DANCR) was up-regulated in CRC. DANCR is an lncRNA acting as an oncogene in various types of cancer including esophageal squamous cell carcinoma, cervical cancer and hepatocellular carcinoma [9–11]. Besides, CRC patients with high DANCR expression tended to have a worse prognosis [12]. DANCR was reported to promote CRC proliferation and metastasis in cultured cells by acting as a molecular sponge of miR-577 [13]. The above data indicate that DANCR plays an important role in the molecular mechanism of CRC and suggest that it may have a potential value for CRC diagnosis. Other than its stability in plasma or serum lncRNAs, DANCR is also closely related to the development and progression of cancer [14–16], showing a promising perspective of serum DANCR as a novel biomarker for the clinical diagnosis of CRC.

The aim of the present study was to observe the expression and clinical significance of serum DANCR in CRC and validate the correlation between the serum DANCR expression and the clinicopathological characteristics of CRC patients in an attempt to establish a new model for the early diagnosis and dynamics monitoring of the progression of CRC.

## Materials and methods

### GEO datasets

To investigate the differential lncRNA/mRNAs between CRC and normal samples, GSE126092 microarray dataset was downloaded from the GEO website (http://www.ncbi.nlm.nih.gov/geo/). These RNA profiles were provided on platform GPL21047 (Agilent-074348 Human LncRNA v64X180K). Ten CRC tissues and their corresponding normal-appearing tissues (NATs) were in GSE126092 profile. The expression of the selected gene was obtained from starBase (http://starbase.sysu.edu.cn/).

### Patient characteristics

Included in the present study were 40 patients with primary CRC, 10 patients with recurrent CRC, 40 patients with colorectal polyps who were admitted to the Affiliated Hospital of Nantong University between January 2017 and October 2018. The diagnosis of CRC patients was histologically confirmed. Forty healthy individuals who underwent routine physical examination in the same hospital during the same period were used as the negative control (NC). The 40 patients who were randomly selected with primary CRC confirmed by pathology had not received radiotherapy or chemotherapy before hospitalization, from whom 10 matched pre- and post-operative samples were collected. Ten patients with recurrence were selected for secondary post-operative admission and were diagnosed with recurrence. The research protocol was approved by the local ethics committee and informed consent was obtained from all patients and subjects.

### Serum RNA extraction

In the present study, the blood samples were from the remaining samples that were used for routine examination. Serum collected from the blood sample was centrifuged at 1000×***g*** for 15 min. RNA was extracted from 400 μl serum using the serum RNA extraction kit (Life Technologies, U.S.A.). Briefly, 200 μl chloroform was added to the serum and centrifuged at 12000 r/min to obtain the supernatant. After addition of 900 μl absolute ethyl alcohol, the sample was placed on RNeasy Mini Spin centrifugal columns and centrifuged. After addition of 700 μl RWT buffer to the centrifuge column, the sample was centrifuged at 12000 r/min, added with 500 μl RPE buffer and centrifuged again at 12000 r/min. After washing and elution, the concentration and purity of the obtained RNA were measured by ultraviolet spectrophotometry. The OD_260_/OD_280_ ratio of RNA samples was calculated, and the ratio between 1.8 and 2.0 indicated that the extracted RNA was of good purity, and the total RNA samples were stored at −80°C for later use.

### cDNA synthesis

Reverse transcription of the extracted RNA was performed using the reverse transcription kit (Thermo Fisher Scientific, U.S.A.) according to the prescribed protocol. Approximately 5 ng of RNA sample and 10 μl of RT master mix were added in the reaction buffer and incubated at 42°C for 60 min and at 72°C for 5 min.

### Real-time PCR

ABI 7500 PCR Detection System (ABI, U.S.A.) was used for real-time PCR. The sequences of DANCR forward and reverse primers were: 5′-GCGCCACTATGTAGCGGGTT-3′ and 5′-TCAATGGCTTGTGCCTGTAGTT-3′, respectively. GAPDH: 5′-TGATGACATCAAGAAGGTGGTGAAG-3′, and 5′-TCCTTGGAGGCCATGTGGGCCAT-3′, respectively. The PCR mixture was composed of 1 μl forward primer, 1 μl reverse primer, 5 μl RNase-free H_2_O, 10 μl SYBR Green I mix and 3 μl cDNA. The amplification procedure was run according to the following steps: incubation at 95°C for 10 min, then product amplification by 40 cycles of 95°C for 15 s and 60°C for 60 s. All assays were operated in triplicate. The comparative *C*_t_ method was utilized for the relative expression calculation.

### Detection of CEA and CA199 levels

MODULAR ANALYTICS E170 (Roche, Germany) was used to detect the concentrations of CEA and CA199 in the sera of the CRC patients in the Laboratory Medicine Center of the Affiliated Hospital of Nantong University. The content of CEA >5 ng/ml or CA199 >37 U/ml was defined as a positive result.

### Statistical analysis

SPSS software, 18.0 (SPSS, Inc., Chicago, IL, U.S.A.) and GraphPad Prism 6.0 (San Diego, CA, U.S.A.) were applied to present the data as medians (lower quartile and upper quartile) of triplicate assay. Spearman coefficient was performed for correlation analysis. The Mann-Whiney *U*-test was used to compare the DANCR of serum DNA between in CRC, colorectal polyps and healthy control groups. Receiver operating characteristic curve (ROC) and the area under the curve (AUC) (95% CI) were used to assess the diagnostic value of DANCR, CEA and CA199 for CRC. Results were considered statistically significant at *P*<0.05 (two-tailed).

## Results

### Identification of lncRNA biomarkers from the training dataset

**Figure 1:**
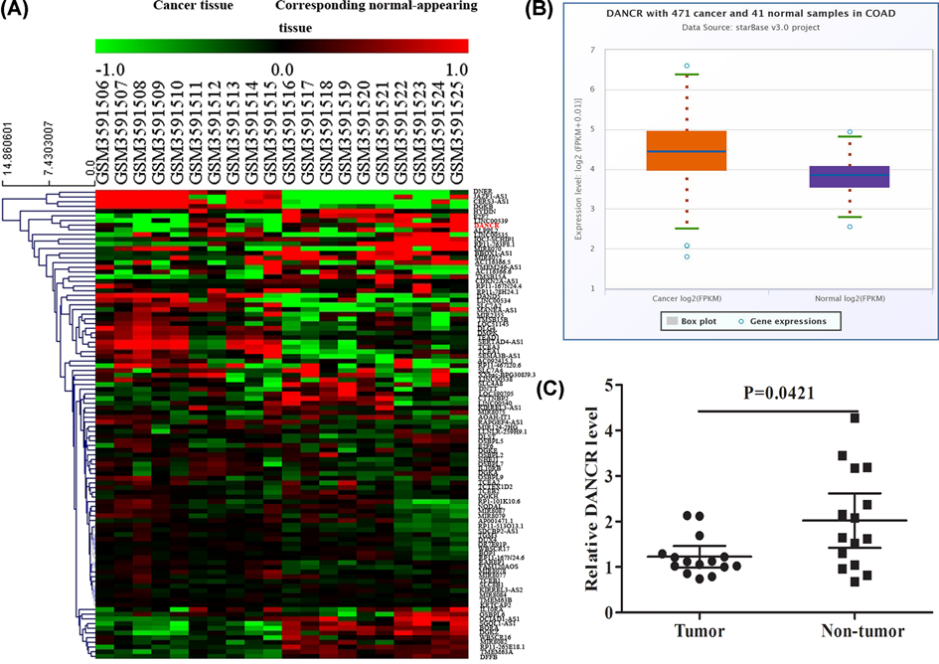
Identification of lncRNA biomarkers from the training dataset (**A**) Some significantly dysregulated lncRNAs/mRNAs in CRC as depicted in the heat map. (**B**) The expression of DANCR in CRC in starBase database. (**C**) Real-time PCR analyzed the expression of DANCR in 15 CRC tissues.

The original CEL files were downloaded and classified as CRC and normal groups. The raw data were standardized and transformed into expression values using the affy package of Bioconductor (http://www.bioconductor.org/). The significance analysis of the empirical bayes methods within limma package was applied to identify differentially expressed genes (DEGs) between the CRC and normal tissues [17]. *P*-value <0.01 and |logFC| > 1 were set as the cut-off criteria to select the significant DEGs. Hierarchical clustering analysis showed a clear distinction on some significantly dysregulated expression of lncRNAs/mRNA in cancer as depicted in the heat map (Figure 1A). Among them, we selected an lncRNA, named DANCR, which was up-regulated in CRC. The data from starBase database that derived from the Cancer Genome Atlas (TCGA) database show that the expression of DANCR in CRC is higher than normal (Figure 1B). To further assess whether DANCR was dysregulated in CRC, we performed real-time PCR to analyze the expression of DANCR in 15 CRC tissues and found that the level of DANCR was significantly increased in the CRC tissue, as compared with the matched adjacent normal tissue (U = 63.0, *P*=0.0421) (Figure 1C).

### Significant increase in serum DANCR in CRC

**Figure 2:**
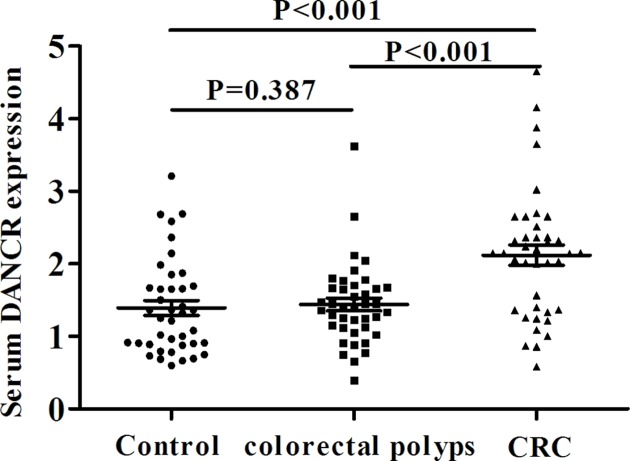
Significant increase of serum DANCR in CRC The relative expression of serum DANCR in CRC, colorectal polyps and healthy control groups.

To analyze its value as a biomarker in CRC, real-time PCR was applied to study the expression of serum DANCR. It was found that the level of serum DANCR in CRC, colorectal polyps and healthy control groups was 2.146 (1.363, 2.477), 1.399 (1.120, 1.671) and 1.287 (0.892, 1.686), respectively. The DANCR expression in CRC group was significantly higher than that in the other two groups (U = 404.0, 407.5, both *P*<0.001), and there was no significant difference between the latter two groups (U = 709.5, *P*=0.387) (Figure 2), suggesting that DANCR was differentially expressed in CRC.

### Correlations between serum DANCR level and clinicopathological features of the CRC patients

Following statistical analysis showed no conspicuous variation in serum DANCR expression in terms of age, gender, grade of tumor differentiation and tumor size in the CRC patients (*P*>0.05), while conspicuous variations were observed among different TNM stages (*P*<0.05) (Table 1).

**Table 1:** Correlations between the relative expression of serum DANCR and clinicopathologic features in CRC patients

Clinicopathologic features	Cases	DANCR relative expression [median (lower quartile, upper quartile)]	U-value	*P*-value
Age (years)			152.5	0.844
≤60	11	2.031 (1.398, 2.366)		
>60	29	2.198 (1.294, 2.584)		
Sex			148.0	0.276
M	25	2.141 (1.348, 2.340)		
F	15	2.301 (1.362, 3.021)		
Tumor size			177.5	0.517
≥5 cm	13	2.166 (1.255, 2.437)		
<5 cm	27	2.147 (1.784, 2.654)		
Differentiation			219.5	0.613
High/intermediate	21	2.198 (1.362, 2.698)		
Low	19	2.147 (1.790, 2.654)		
TNM			101.5	0.019*
I, II	29	2.141 (1.294, 2.338)		
III	11	2.654 (2.023, 3.906)		

*P<0.05.

### Correlations between DANCR expression and existing CRC diagnostic indicators

**Figure 3:**
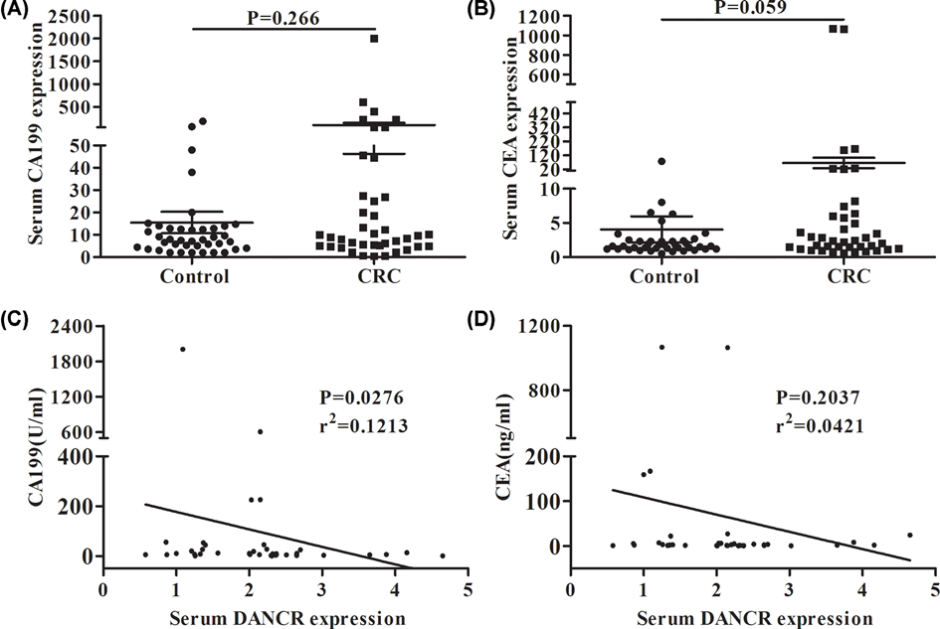
Correlations between the expression of DANCR and CEA and CA199 The relative expression of CA199 (**A**) and CEA (**B**) in CRC and healthy control groups. Correlations between the relative expression of serum DANCR and CA199 (**C**), CEA (**D**) in CRC patients.

To evaluate the clinical utility of DANCR, we evaluated several existing diagnostic indicators including serum CEA and CA199 in the CRC patients and found no significant difference in serum CEA and CA199 between the CRC patients and the normal controls (*P*>0.05) (Figure 3A,B). Then we analyzed the correlations between DANCR and the above two indicators in CRC. The results showed that DANCR expression was significantly correlated with the CA199 concentration (r^2^ = 0.1213, *P*=0.0276) (Figure 3C), but not with the CEA concentration (r^2^ = 0.0421, *P*=0.2037) (Figure 3D).

### Diagnostic accuracy of serum DANCR in CRC

**Figure 4:**
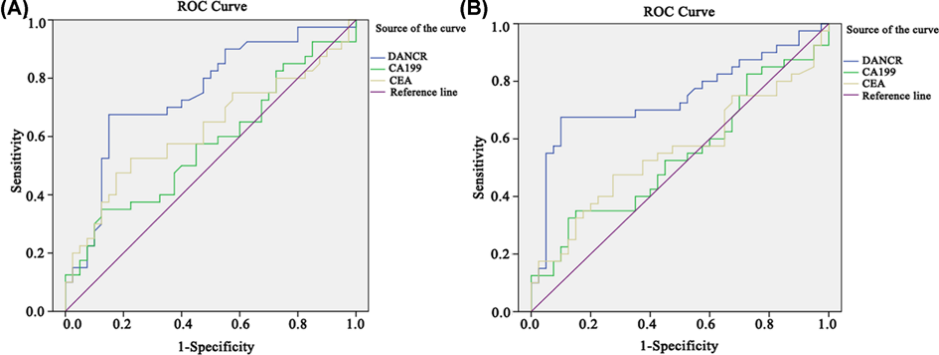
Diagnostic accuracy of the serum DANCR ROC curves of DANCR, CEA and CA199 for differentiating CRC patients from healthy control group (**A**) and colorectal polyps group (**B**).

ROC curves of DANCR, CEA and CA199 in CRC, colorectal polyps and NC groups were mapped. In terms of comparison between CRC and NC groups, the related sensitivity and specificity of DANCR were 67.5 and 82.5% with the cut-off value setting at 1.994. As for CEA and CA199, the sensitivity was 40.0 and 32.5% and the specificity was 85 and 80.0%, respectively. The AUC of DANCR was 0.747 (95% CI: 0.638–0.857), while 0.623 for CEA (95% CI: 0.498–0.748) and 0.573 for CA199 (95% CI: 0.446–0.699) (Figure 4A). To see whether DANCR was able to distinguish between CRC and colorectal polyps, we compared the two groups and found that at the cut-off value of 1.956, the related sensitivity and specificity of DANCR were 67.5 and 87.5%, respectively with the AUC of 0.745 (95% CI: 0.632–0.859) *vs.* 0.555 for CEA (95% CI: 0.427–0.684) and 0.542 for CA199 (95% CI: 0.413–0.670) (Figure 4B). All these findings suggest that DANCR seemed better than CEA and CA199 in terms of the diagnostic value for CRC.

### Combination of DANCR, CEA and CA199 to construct a novel diagnostic model

**Figure 5:**
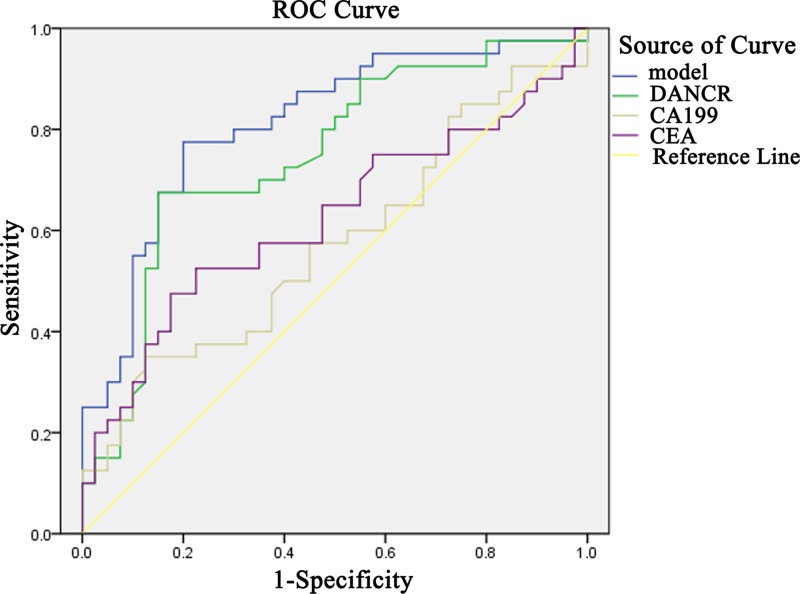
Simultaneous detection of DANCR, CEA and CA199 to construct a novel diagnostic model ROC analysis of individual or simultaneous detection of DANCR, CEA and CA199 in differentiating CRC from healthy controls.

Using the healthy group as the control, some diagnostic test evaluation indicators were calculated in DANCR-, CEA- or CA199-alone group, two-combination group or three-combination group to assess their sensitivity, specificity, accuracy, the positive predictive value and the negative predictive value. It was found that the sensitivity and negative predictive values of the three-combination group were 87.5 and 81.5%, respectively, which were the highest of the three groups (Table 2). Next, the logistic regression was used to explore the diagnostic efficiency when the three serum biomarkers were combined at the same time. As shown in Figure 5, the model combining DANCR, CEA and CA199 yielded a good diagnostic efficacy for CRC patients with an AUC of 0.812, which was higher than that of the two-combination group or either of the three indicators alone.

**Table 2:** Diagnostic efficacy of serum DANCR, CEA and CA199 in differentiating CRC patients from healthy control group

Molecular marker	Sensitivity	Specificity	Curacy	Positive prediction	Negative prediction
	(%)	(%)	(%)	(%)	(%)
DANCR	67.5	82.5	75.0	79.4	71.7
	(27/40)	(33/40)	(60/80)	(27/34)	(33/46)
CEA	40.0	85.0	62.5	72.7	58.6
	(16/40)	(34/40)	(50/80)	(16/22)	(34/58)
CA199	32.5	80.0	56.2	61.9	54.2
	(13/40)	(32/40)	(45/80)	(13/21)	(32/59)
DANCR+CEA	80.0	70.0	75.0	72.7	77.7
	(32/40)	(28/40)	(60/80)	(32/44)	(28/36)
DANCR+CA199	77.5	65.0	71.3	68.9	74.3
	(31/40)	(26/40)	(57/80)	(31/45)	(26/35)
Combined detection of the three markers	87.5	55.0	71.3	66.0	81.5
	(35/40)	(22/40)	(57/80)	(35/53)	(22/27)

### Utility of monitoring tumor dynamics in CRC patients

**Figure 6:**
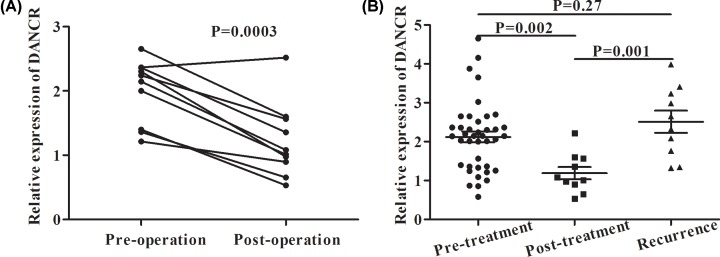
Utility of DANCR in monitoring tumor dynamics of CRC patients (**A**) Ten pairs of pre- and post-operative samples were collected to detect DANCR expression. (**B**) DANCR expression was detected in 40 primary CRC patients, 10 patients after chemotherapy or surgery treatment and 10 patients with tumor recurrence.

To observe the dynamic change in DANCR expression in the CRC patients after surgery, ten matched pre- and post-operative CRC samples were collected. As shown in Figure 6A, serum DANCR was dynamically decreased after surgical treatment (*P*<0.05). We also compared the DANCR expression in 40 primary CRC patients, 10 patients after chemotherapy or surgical treatment and 10 patients with tumor recurrence, and found serum DANCR expression was significantly lower in the post-treatment patients than that in the pre-treatment patients and recurrent patients (*P*<0.05) (Figure 6B). The observations were suggestive of the utility of DANCR in monitoring tumor dynamics in CRC patients.

## Discussion

Despite advances in screening detection and new treatment strategies, CRC remains a leading cause of cancer-associated mortality, partly due to the lack of effective early diagnostic methods and reduced the sensitivity to chemotherapy [18]. Therefore, it is necessary to develop more sensitive and specific diagnostic and prognostic biomarkers and therapeutic targets for CRC.

With advances in RNA sequencing (RNA-seq) and microarray profiling techniques, a large number of abnormally expressed lncRNAs have been identified in various cancers [19]. Huang et al. [20] identified hundreds of CRC-associated DEGs based on the GEO and TCGA database. An et al. [21] found 35 genes that were significantly associated with patient survival based on the transcription profile of multistage carcinogenesis and bioinformatics analysis. Tissue and serum lncRNAs have been studied as novel diagnostic and prognostic biomarkers in cancer in recent years [22–27].

Previous studies have shown that lncRNAs function as oncogenes or tumor suppressors in association with tumorigenesis of many malignancies, including CRC [28–30]. Hence, expounding the detailed molecular mechanisms of lncRNAs is urgently required. In the present study, we identified DANCR from GSE126092 microarray dataset including differential lncRNA/mRNAs between CRC and normal tissue samples, knowing that dysregulation of DANCR may affect the proliferation, differentiation and apoptosis of many types of cancer cells, including CRC [9]. Given the importance of lncRNAs in biological processes and the few studies reporting the clinical significance of serum DANCR in CRC patients, we focused on serum DANCR expression and its diagnostic value in CRC in the present study.

We first demonstrated that serum DANCR was up-regulated in CRC patients, which is consistent with previous studies [13]. In addition, we found that serum DANCR instead of tissue DANCR could be used for differential diagnosis between CRC, colorectal polyps and healthy controls. To evaluate the diagnostic efficacy of DANCR, ROC curve was performed, yielding an AUC of 0.747, a good sensitivity (67.5%) and a good specificity (82.5%).

The clinicopathological parameters showed that high serum DANCR was associated with TNM stages, indicating a tendency of increased serum DANCR expression in the advanced stage of CRC patients. Additionally, the serum DANCR expression level in patients after surgery or chemotherapy treatment decreased significantly to a level similar to that in the healthy controls, but rebounded in cases of tumor recurrence, suggesting that serum DANCR could be used to monitor disease progression and predict prognosis of CRC patients.

Although the relative expression of DANCR was significantly correlated with CA199 but not with CEA, we constructed a model of simultaneous detection of the three biomarkers to improve the diagnostic efficiency. The AUC of our model was 0.812, which was higher that the two-combination protocol, or either of the three indicators alone, suggesting that serum DANCR may prove to be a useful marker in auxiliary diagnosis of CRC.

Nevertheless, there are still some limitations in this work. First, the sample size of the present study is relatively small and the results were obtained from a single center. In addition, the conclusion of the present study needs to be confirmed in larger sample and multicenter studies, and the mechanism of DANCR in CRC needs to be further explored.

In summary, our study demonstrated that serum DANCR was elevated in CRC patients, which may aid CRC diagnosis and tumor dynamics monitoring. Simultaneous detection of serum DANCR, CEA and CA199 could improve the diagnostic efficacy of CRC. DANCR may prove to be an important biomarker of CRC.

## Abbreviations

AUC: area under the curve
CEA: carcinoembryonic antigen
CI: confidence interval
CRC: colorectal cancer
DANCR: differentiation antagonizing non-protein coding RNA
DEG: differentially expressed gene
GEO: Gene Expression Omnibus
lncRNA: long non-coding RNA
ROC: receiver operating characteristic curve
TCGA: the Cancer Genome Atlas
